# High Log-Scale Expansion of Functional Human Natural Killer Cells from Umbilical Cord Blood CD34-Positive Cells for Adoptive Cancer Immunotherapy

**DOI:** 10.1371/journal.pone.0009221

**Published:** 2010-02-15

**Authors:** Jan Spanholtz, Marleen Tordoir, Diana Eissens, Frank Preijers, Arnold van der Meer, Irma Joosten, Nicolaas Schaap, Theo M. de Witte, Harry Dolstra

**Affiliations:** 1 Laboratory of Hematology, Department of Laboratory Medicine, Nijmegen Centre for Molecular Life Sciences, Radboud University Medical Centre, Nijmegen, The Netherlands; 2 Laboratory of Medical Immunology, Department of Hematology, Nijmegen Centre for Molecular Life Sciences, Radboud University Medical Centre, Nijmegen, The Netherlands; 3 Laboratory of Medical Immunology, Department of Laboratory Medicine, Nijmegen Centre for Molecular Life Sciences, Radboud University Medical Centre, Nijmegen, The Netherlands; Centre de Recherche Public de la Santé (CRP-Santé), Luxembourg

## Abstract

Immunotherapy based on natural killer (NK) cell infusions is a potential adjuvant treatment for many cancers. Such therapeutic application in humans requires large numbers of functional NK cells that have been selected and expanded using clinical grade protocols. We established an extremely efficient cytokine-based culture system for *ex vivo* expansion of NK cells from hematopoietic stem and progenitor cells from umbilical cord blood (UCB). Systematic refinement of this two-step system using a novel clinical grade medium resulted in a therapeutically applicable cell culture protocol. CD56^+^CD3^−^ NK cell products could be routinely generated from freshly selected CD34^+^ UCB cells with a mean expansion of >15,000 fold and a nearly 100% purity. Moreover, our protocol has the capacity to produce more than 3-log NK cell expansion from frozen CD34^+^ UCB cells. These *ex vivo*-generated cell products contain NK cell subsets differentially expressing NKG2A and killer immunoglobulin-like receptors. Furthermore, UCB-derived CD56^+^ NK cells generated by our protocol uniformly express high levels of activating NKG2D and natural cytotoxicity receptors. Functional analysis showed that these *ex vivo*-generated NK cells efficiently target myeloid leukemia and melanoma tumor cell lines, and mediate cytolysis of primary leukemia cells at low NK-target ratios. Our culture system exemplifies a major breakthrough in producing pure NK cell products from limited numbers of CD34^+^ cells for cancer immunotherapy.

## Introduction

Natural Killer (NK) cells are CD56^+^CD3^−^ large granular lymphocytes that exert innate immunity against viral infections and cancer [1]. NK cells recognize and subsequently react to virus-infected and transformed cells without prior immunization, basically through the balance of signals from inhibitory and activating receptors [2]. Therefore, NK cells have been previously described as promising effectors for adoptive immunotherapy against cancer [3]. The anti-tumor potential of NK cells has been best demonstrated during therapy of leukemia patients with allogeneic stem cell transplantation (SCT). Ruggeri et al. demonstrated that NK cell alloreactivity can control relapse of acute myeloid leukemia (AML) without causing graft-versus-host disease (GVHD) in the setting of HLA-mismatched haploidentical allogeneic SCT [4]. In addition, haploidentical NK cell infusions together with IL-2 in a non-transplantation setting have been associated with complete hematologic remission in poor-prognosis patients with AML [5]. These encouraging results point out that allogeneic NK cell-based immunotherapy may be a promising therapeutic strategy for AML in both the non-transplant and post-transplant setting [5], [6].

To date, most clinical studies exploiting allogeneic NK cells for adoptive immunotherapy have been performed with NK cells selected from leukapheresis products by immunomagnetic beads selection protocols [7]–[12]. In order to circumvent limitations in cell numbers, purity and state of activation of such blood-derived NK cells, *ex vivo* expansion of NK cells with higher purity will facilitate the infusion of a greater number of activated NK cells in patients with a relatively large tumor burden or permits multiple NK cell infusions [8], [13], [14]. Therefore, development of innovative strategies enabling the generation of clinically relevant NK cell products with high cell numbers, high purity and functionality promises a major breakthrough in NK cell-based immunotherapy.

In this study, we developed a cytokine-based method for large scale expansion of functional CD56^+^ NK cells from hematopoietic stem and progenitor cells. Similar studies have been performed previously focusing on either CD34^+^ hematopoietic progenitor cells (HPC) from bone marrow (BM) or umbilical cord blood (UCB) [15]–[17]. However, most of these culture systems are unsuitable for clinical application because of the use of animal sera, animal-derived proteins and supportive feeder cell lines. Furthermore, most of these methods yielded only limited NK cell numbers for successful adoptive immunotherapy in cancer patients. In order to surmount these shortcomings, we established a two-step culture scheme in which we used a novel clinical grade medium to generate more than 3 to 4-log fold-expanded functional CD56^+^ NK cells from freshly selected or cryopreserved CD34^+^ UCB cells, respectively. The CD56^+^ NK cell products generated by this method have a very high purity, contain various NKG2A and killer immunoglobulin-like receptor (KIR) expressing mature subsets and efficiently lyse AML and solid tumor cells. These findings exemplify that this culture system could hold great promise for the *ex vivo* generation of clinical grade NK cell products for cellular immunotherapy against cancer.

## Results

### *Ex vivo* Progenitor Cell Expansion and NK Cell Differentiation

**Figure 1:**
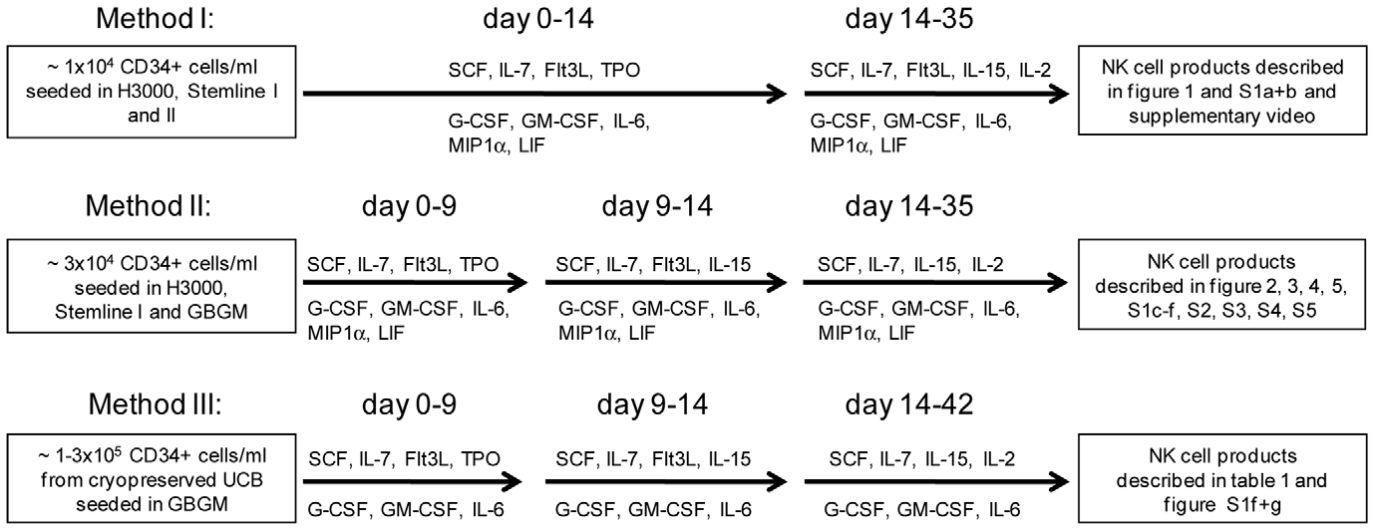
Schematic diagram of the different culture methods used for the ex *vivo* generation of CD56^+^ NK cells from cytokine-expanded CD34^+^ UCB cells. In Method I–III different combinations of cytokines were tested as described in detail in Materials and Methods starting with different numbers of initially seeded CD34^+^ cells. In Method I and II, NK cells were generated from freshly selected UCB donors and the culture duration was 5 weeks. In Method III, CD34^+^ cells were used from cryopreserved UCB donors and the culture period was 6 weeks. Finally, the diagram depicts which NK products were used and displayed as results in figures and supplemental material.

The aim of this study was to develop an efficient cytokine-based *ex vivo* culture system for the expansion of CD34^+^ cells followed by the subsequent log-scale generation of CD56^+^CD3^−^ NK cells. To identify a suitable medium for clinical applicable expansion and differentiation of NK cells, we tested different basal media using a two-step *in vitro* differentiation scheme (Figure 1). Initially, we compared the media H3000, Stemline I and Stemline II seeding 1×10^4^ CD34^+^ cells using Method I. We detected a strong increase in CD34^+^ cell numbers in all three media, resulting in a mean expansion rate for all experiments (n = 15) of 48±7 fold and 78±27 fold after 1 and 2 weeks of culture, respectively. The total cell expansion was associated with a gradual decline of the frequency of CD34^+^ cells from 84%±16% at day 0 till 47%±14% at week 1 and 17%±9% at week 2 (Figure S1a and S1b).

**Figure 2:**
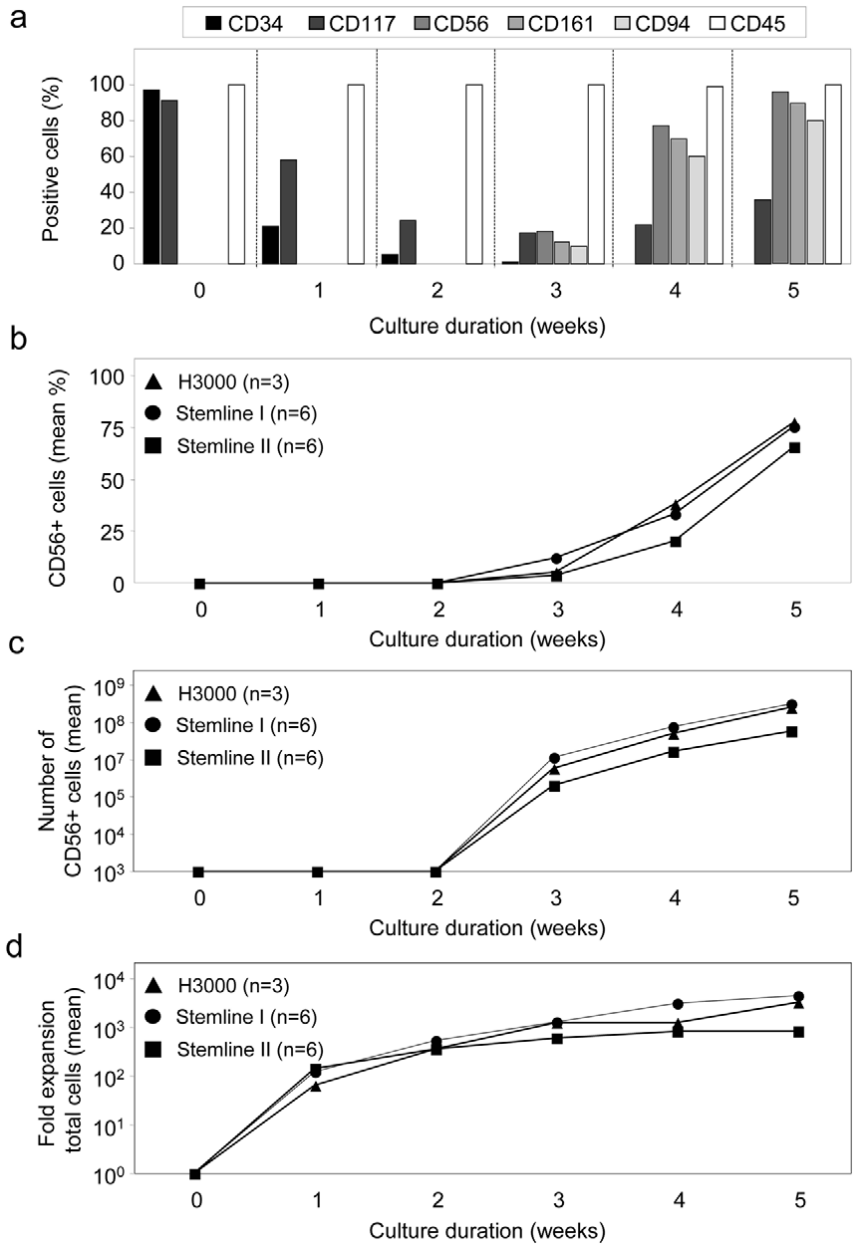
*Ex vivo* generation of CD56^+^ NK cells from cytokine-expanded CD34^+^ UCB cells. CD34-enriched UCB cells were expanded for two weeks using three different media (H3000, Stemline I and Stemline II) and subsequently differentiated into NK cells for three additional weeks in the same basal medium using Method I (see Figure 1). Cell cultures were weekly analyzed for cell numbers and phenotype using FCM. (**a**) Representative example of antigen expression during the two-step culture period using H3000 medium. One week after the onset of the NK cell differentiation step 2 (i.e. after 3 weeks total culture duration) the CD56^+^CD161^+^CD94^+^ NK cell population increases and reaches high purity after 3 weeks of differentiation (i.e week 5). (**b**) Mean CD56^+^ cell frequency during the 5 week culture period for three different media, which have been tested in parallel experiments using 3–6 UCB donors. (**c**) Mean total CD56^+^ NK cell numbers after initial seeding of 1×10^4^ CD34^+^ UCB cells during 5 weeks of culture using Method I. Data represent a theoretical calculation based on the actual expansion rates of CD56^+^ cells. Total yield of CD56^+^ cells at each week was calculated by multiplying the expansion rate per week with the number of cultured cells. (**d**) Mean fold expansion of total cells after initial seeding of 1×10^4^ CD34^+^ UCB cells during 5 weeks of culture using Method I. Data represent a theoretical calculation based on the actual expansion rates of total cells.

Next, we investigated whether the expanded UCB-derived CD34^+^ cells were able to differentiate into CD56^+^ NK cells. The differentiation step was monitored by the analysis of the cell surface molecules CD34, CD117, CD56, CD94 and CD161, which have been described to be expressed at different human NK cell developmental stages *in vivo* [18]. We observed after 3 weeks total culture duration that the percentage of CD34^+^ cells further declined, while the CD56^+^CD161^+^CD94^+^ NK cell population increased to 10–18% (e.g. Figure 2a). Thereafter, the population of CD56^+^CD161^+^CD94^+^ cells rapidly increased to 60–77% after 4 weeks and 80–96% after 5 weeks of culture (e.g. Figure 2a).

Although we could not detect significant differences between the different basal media, the purity of the CD56^+^ cell product appeared slightly higher with H3000 medium (77%±24%; n = 3) and Stemline I medium (75%±21%; n = 6) as compared to Stemline II medium (66%±17%; n = 6) (Figure 2b). Furthermore, a trend was observed towards higher CD56^+^ cell numbers with Stemline I medium (range 4×10^6^–1.1×10^8^ CD56^+^ cells; n = 6) followed by H3000 (5.2×10^6^–5.4×10^7^ CD56^+^ cells; n = 3) and Stemline II medium (range 1×10^6^–2×10^7^ CD56^+^ cells; n = 6) (Figure 2c). The mean total cell expansion after 5 weeks of culture with Stemline I medium was ∼4,400-fold and with H3000 medium ∼3,200-fold, but only ∼850-fold expansion with Stemline II (Figure 2d). These results indicate that the culture conditions support an effective outgrowth of CD56^+^ NK cells with a high purity of the final NK cell product after a culture period of 5 weeks using various basal media.

### Superior Expansion of Extremely Pure NK Cell Products Was Achieved Using a Novel Clinical Grade Medium

**Figure 3:**
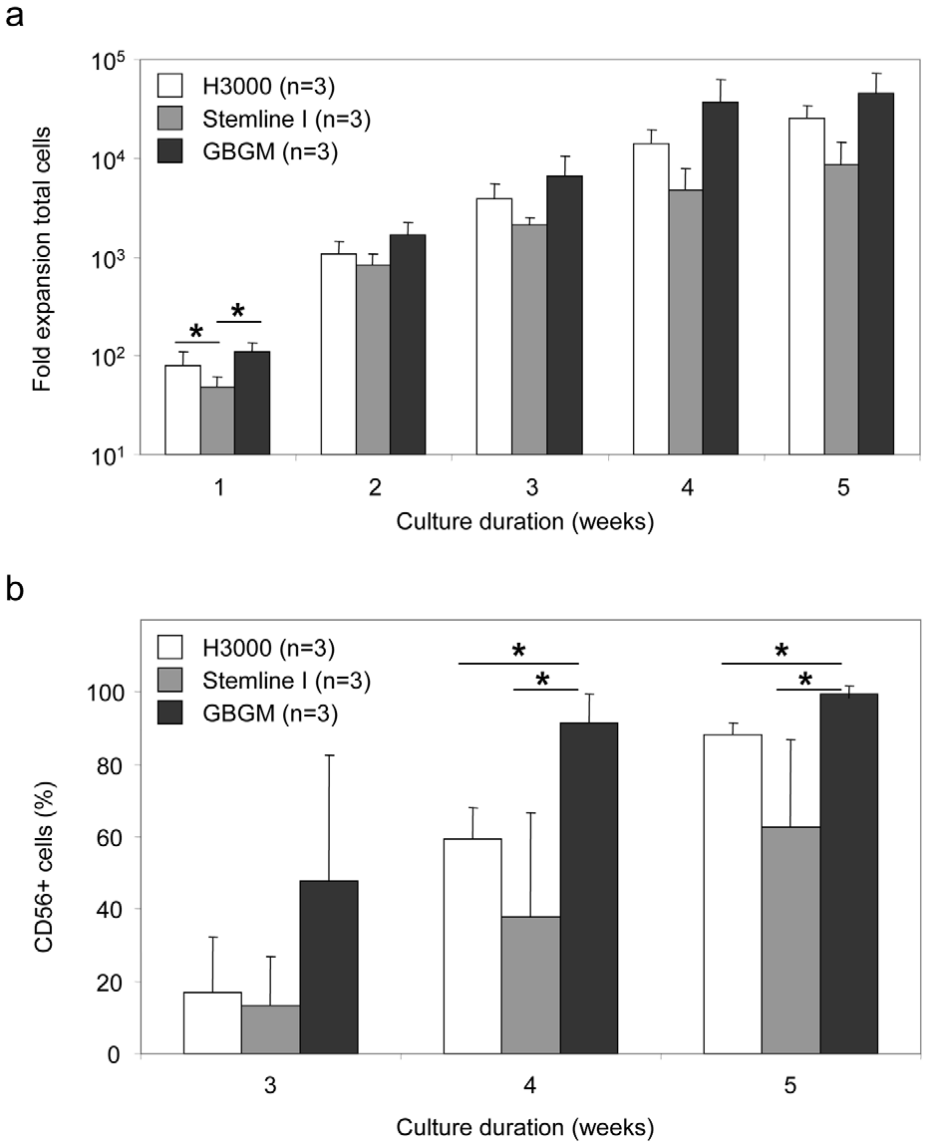
Superior expansion of UCB-derived CD56^+^ NK cells using a novel clinical grade medium. The new GBGM® medium was compared with two previously tested media in the NK cell generation system according to Method II (see Figure 1). Cell cultures were weekly analyzed for cell numbers and phenotype using FCM. (**a**) Fold expansion of total cells after initial seeding of 1×10^4^ CD34^+^ UCB cells was determined during 5 weeks of culture. Data represent the calculation based on the actual expansion rates of total cells and are displayed as mean ± SD of three different experiments. (**b**) CD56^+^ cell frequency during the differentiation stage for three different media, which have been tested in parallel experiments using three UCB donors. Data are depicted as mean ± SD. * represents a p-value of <0.05.

Although high expansion rates and purities could be obtained with current commercially available basal media, we were not satisfied with the purity of the CD56^+^ NK cell product following 5 weeks of culture using Method I (Figure 1). To further increase the efficiency of our culture method, we tested a newly formulated serum-free medium, designated Glycostem Basal Growth Medium (GBGM®), which is produced under GMP conditions and suitable for clinical applications. In addition, we slightly adjusted the cytokine conditions during the expansion step, since we observed that expansion of CD34^+^ cells as well as total cells was most prominent during the first week of culture (Figure S1a and S1c). Furthermore, to increase the balance towards expansion of NK cell progenitors we added IL-15 instead of TPO to the expansion medium from day 9 of culture, and left out Flt3L in the differentiation medium (Figure 1). Using this modified Method II, culture in GBGM® resulted in the highest total cell expansion during the first week and subsequent differentiation into CD56^+^ NK cells (Figure 3 and Figure S1e). The total cell expansion rate in GBGM® increased to >15,000 fold (range 16,991–73,666; n = 3) (Figure 3a). More importantly, *ex vivo* generation of CD56^+^ NK cells in GBGM® yielded a significant higher purity of 99%±1% (n = 3) compared to H3000 with 88%±3% (n = 3; p<0.05) and Stemline I with 63%±24% (n = 3; p<0.05), respectively (Figure 3b). These data demonstrate that the inventive modified culture system using the newly formulated GBGM® medium results in more than 4-log expansion of pure human NK cells from freshly selected CD34^+^ UCB cells.

### Phenotypic Profile of CD56^+^ NK Cells Derived from Expanded CD34^+^ Cells

**Figure 4:**
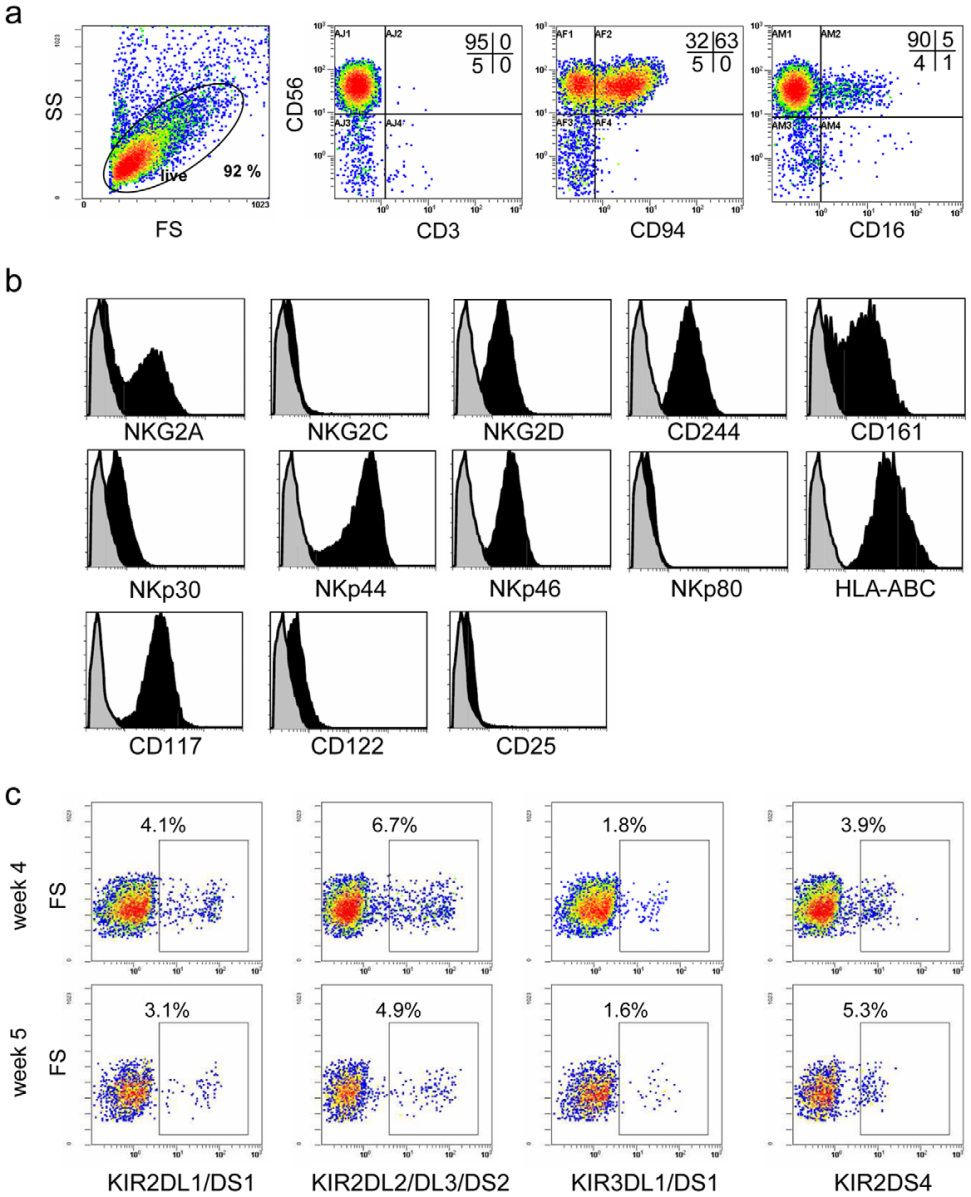
Phenotypical profile of *ex vivo*-generated NK cells using Method II with GBGM. (**a**) Flow cytometric analysis of a representative NK cell product generated from CD34^+^ UCB progenitor cells. Cells at 5 weeks of culture were analyzed for expression of CD56, CD3, CD94 and CD16. (**b**) Expression of a repertoire of receptors important for regulating NK cell activity, including C-type lectin receptors, natural cytotoxicity receptors and cytokine receptors. Histograms show expression of the antigen of interest (black histogram) compared to the specific isotype control (grey histogram). (**c**) Acquisition of KIR^+^ NK cell subsets during *ex vivo* NK cell generation from expanded CD34^+^ UCB cells. KIR expression was determined at week 4 and 5 during the differentiation step by FCM.

Flow cytometric analysis revealed that the *ex vivo*-generated NK cells after 5 weeks of culture contained a homogeneous cell population displaying high expression of CD56 in the absence of CD3 (Figure 4a). A high frequency of this CD56^+^CD3^−^ NK cell population displayed expression of CD94, while only a limited subset was positive for CD16. Furthermore, NK cell products displayed homogeneous and relatively high expression of NKG2D and the natural cytotoxicity receptors (NCR) NKp30, NKp44 and NKp46, whereas NKG2A was more differentially expressed (Figure 4b and Figure S2a). Additionally to these findings we detected high expression of 2B4 (CD244) and NKR-P1 (CD161) as typical NK cell receptors, while NKG2C and NKp80 were absent or expressed at relatively low levels. In addition, we observed high expression of MHC class I (HLA-ABC) and cytokine receptor chains for IL-2 and IL-15 (CD122; IL-2/IL-15Rβ) and SCF (CD117; c-kit-R), which are important for NK cell differentiation, expansion and activation.

KIR repertoire analysis showed significant individual differences in the frequency of KIR-expressing NK cell subsets between various UCB donors. KIR^+^ NK cell subsets could already be detected at week 4 of culture and remained relatively stable until the end of the culture period at week 5 (Figure 4c and Figure S2b). While some CD56^+^ NK cell products contained positive cells for all four KIR phenotypes analyzed (Figure 4c), others specifically lacked the KIR2DL1/DS1^+^ subset (Figure S2b). Generally, the KIR2DL2/DL3/DS2^+^ subset was present in a higher proportion in all NK cell products analyzed so far, which is in agreement with earlier observations that recovery of KIR2DL2/DL3/DS2^+^ NK cells is faster compared to the KIR2DL1/DS1^+^ subset following HSCT [19].

Taken together, these results illustrate that UCB-derived NK cells generated with our optimized culture system contain developmentally mature NK cell populations expressing NKG2A, KIR and various activating receptors.

### *Ex vivo*-Generated CD56^+^KIR^+^ and CD56^+^NKG2A^+^ NK Cells Are Functionally Regulated by MHC Class I Expression

**Figure 5:**
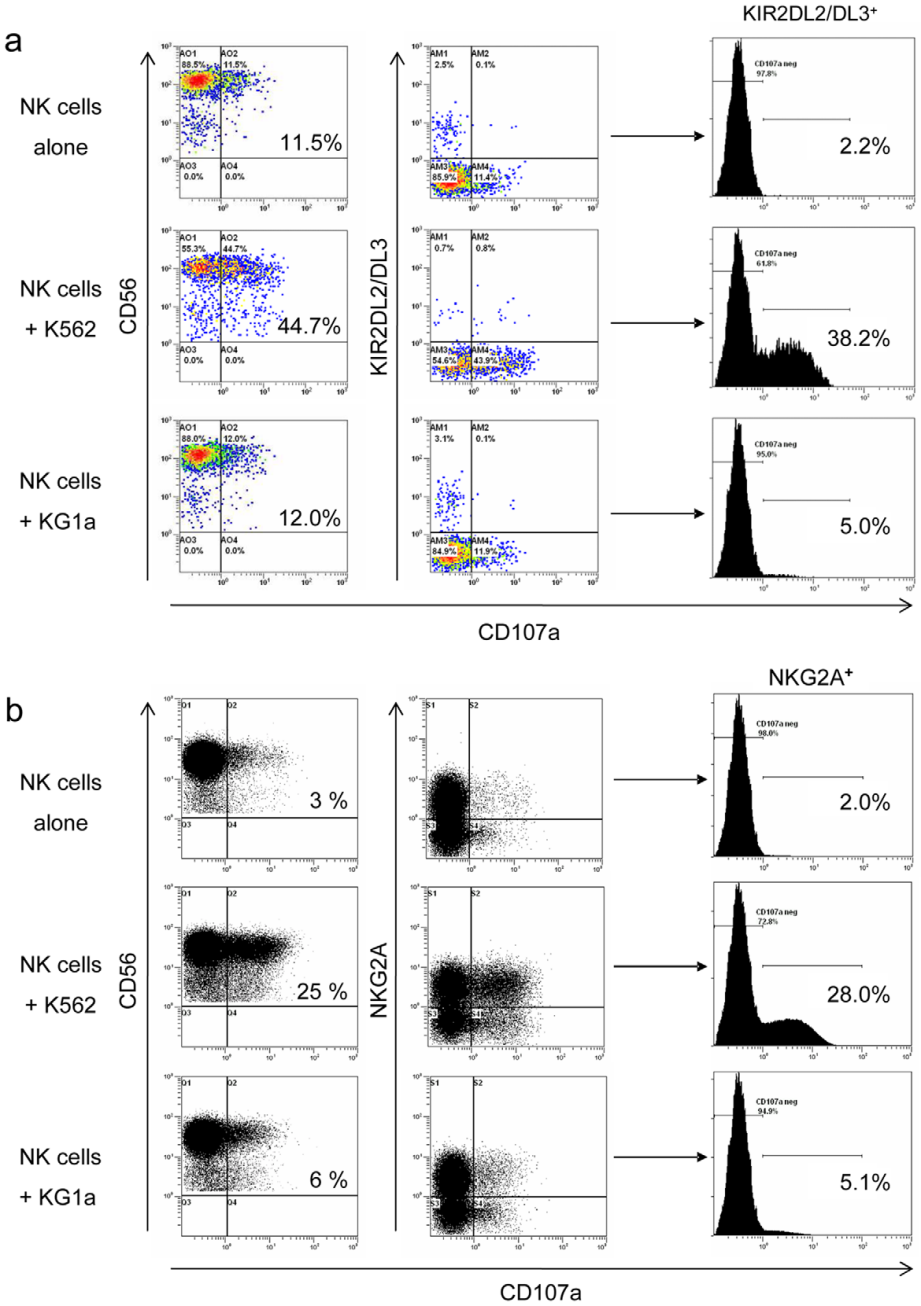
Responsiveness of *ex vivo*-generated KIR^+^ and NKG2A^+^ NK cells to MHC class I-deficient target cells. *Ex vivo*-generated NK cells using Method II with GBGM were incubated alone, or 18 hours with MHC class I-negative K562 or MHC class I-expressing KG1a cells at an E∶T ratio of 1∶1. Cells were then stained for CD56, CD3, KIR or NKG2A, and the degranulation antigen CD107a. (**a**) Degranulation of total CD56^+^CD3^−^ NK cells and KIR2DL2/DL3^+^ NK cell subset expanded for 5 weeks from CD34^+^ UCB cells. Density plots are gated on CD56^+^CD3^−^ NK cells and the histogram plots show the CD107a degranulation of the KIR2DL2/DL3^+^ NK cells. (**b**) Degranulation of total CD56^+^CD3^−^ NK cells and NKG2A^+^ NK cell subset expanded for 6 weeks from CD34^+^ UCB cells. Density plots are gated on CD56^+^CD3^−^ NK cells and the histogram plots show the CD107a degranulation of the NKG2A^+^ NK cells.

Phenotypic analysis showed that our *ex vivo*-generated NK cell products contained up to 10% CD56^+^KIR^+^ cells and a high proportion (40–60%) CD56^+^NKG2A^+^ cells. To determine cytolytic activity of these subsets and investigate if this activity is regulated by the expressed inhibitory receptors, we performed CD107a-based degranulation assays using HLA-negative K562 cells and KG1a cells expressing relatively high levels of HLA-ABC and -E (see Table 1). For these experiments, we used two *ex vivo*-generated NK cell products that contained approximately 15–30% CD56^+^CD107a^+^ cells upon co-culture with K562 (Figure 5 and Figure S3). In contrast, KG1a cells hardly stimulated degranulation of these NK cell products, indicating that cytolytic activity is inhibited by HLA expression. Similarly, around 35% of the CD56^+^KIR2DL2/DL3^+^ subset degranulated upon triggering by K562, while only a small percentage (<5%) expressed CD107a following co-culture with KG1a (Figure 5a and Figure S3). In agreement with these results, degranulation by the CD56^+^KIR2DL2/DL3^+^ subset could be specifically inhibited by K562 cells transfected with the HLA-C group 1 allele HLA-Cw3, while transfection of the HLA-C group 2 allele HLA-Cw4 allele had not effect (Figure S4). Finally, we observed that also the dominant CD56^+^NKG2A^+^ subset was able to efficiently degranulate in response to K562 (28% CD107a^+^ cells), while degranulation towards KG1a was low probably due to relative high expression of HLA-E molecules (Figure 5b and Table 1). These data demonstrate that the KIR^+^ and NKG2A^+^ subsets within the UCB-derived NK cell products are fully responsive mediating strong degranulating activity, which is regulated by the expression of MHC class I molecules on the engaged target cells.

**Table 1:** HLA-C subtype, KIR group and cell surface expression of HLA molecules by the different target cell lines.

Cell line	HLA-Cw typing^1^	KIR group	HLA-ABC^2^	HLA-E^2^
K562	Cw*03, Cw*05	C1 & C2	<1	2
KG1a	Cw*04, Cw*16	C1 & C2	101	5
Lama	Cw*05, Cw*12	C1 & C2	73	5
Kasumi	Cw*03, Cw*08	C1	9	<1
BLM	Cw*07	C1	121	<1
FM3	Cw*05	C2	132	6

1 HLA-Cw typing was performed by SSO-PCR using sequence specific primers according to ASHI guidelines.

2 Expression of HLA molecules was measured by FCM and expressed as delta MFI ( =  MFI specific antibody - MFI isotype control). HLA class A and B types of the cell lines were: K562 (A*11, A*31, B*18, B*40), Lama (A*02, A*25, B*18, B*44), Kasumi (A*26, B*40, B*48), KG1a (A*30, B*53, B*78), BLM (A*02, B*07) and FM3 (A*02, B*44).

### *Ex vivo*-Generated NK Cells Efficiently Target Leukemia and Solid Tumor Cells

**Figure 6:**
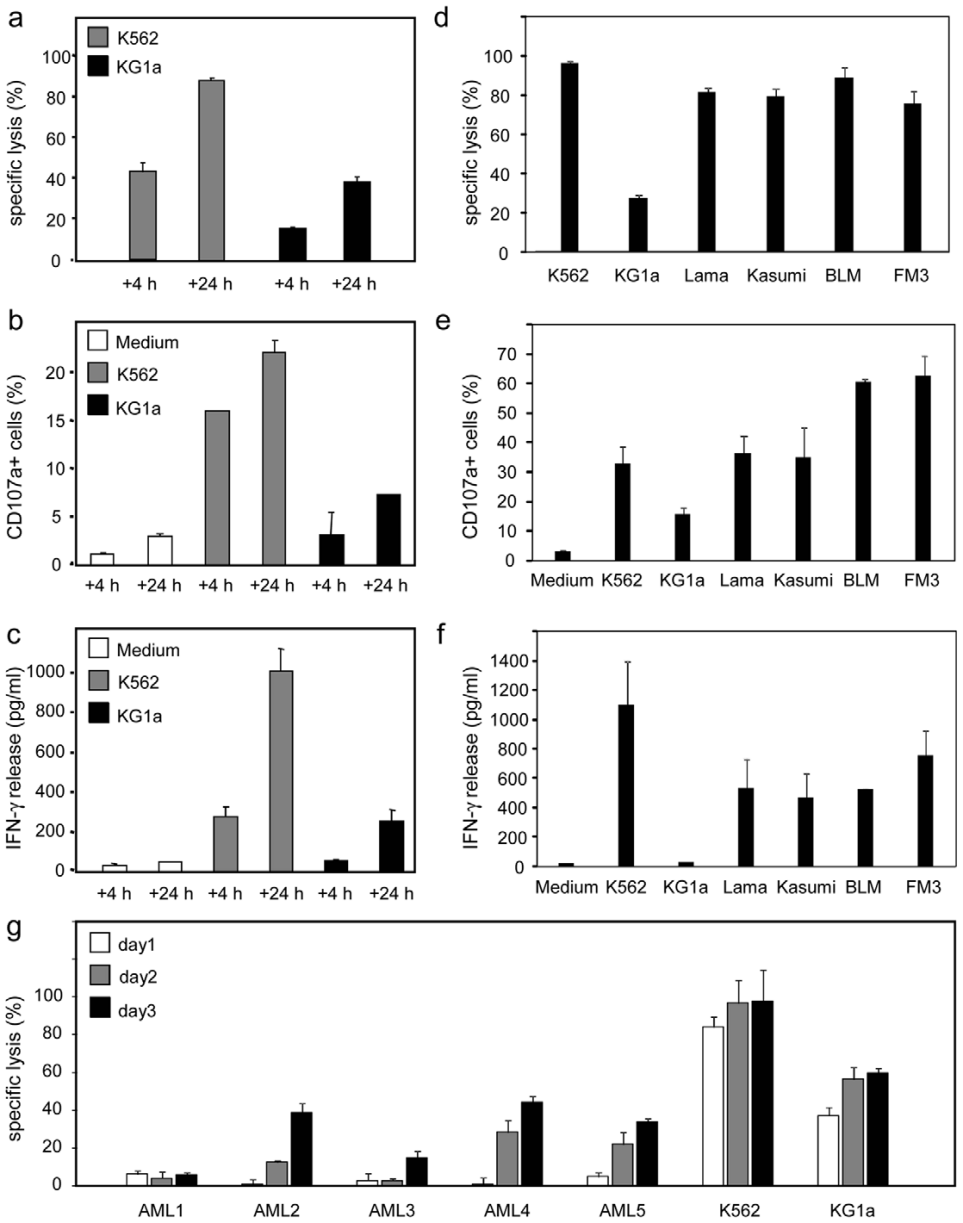
Functional activity of *ex vivo*-generated CD56^+^ NK cells using Method II with GBGM. (**a**) Specific cytotoxicity of a CD56^+^ NK cell product (98% purity) against the myeloid leukemia cell lines K562 and KG1a. Specific lysis was determined after 4 and 24 hours of co-culture in a FCM-based cytotoxicity assay at an E∶T ratio of 2∶1. Data are displayed as mean ± SD of triplicate wells. (**b**) Degranulation of CD56^+^ NK cells was determined by FCM as the percentage of CD107a^+^ cells. Results are depicted as mean ± SD of triplicate wells. (**c**) IFNγ production was determined by ELISA and depicted as mean ± SD of triplicate measurements. (**d**) Specific cytotoxicity of another CD56^+^ NK cell product (95% purity) against the myeloid leukemia cell lines K562, Lama, Kasumi and KG1a, and the melanoma cell lines BLM and FM3. Specific lysis was determined after 18 hours of co-culture in a FCM-based cytotoxicity assay at an E∶T ratio of 1∶1. Data are displayed as mean ± SD of triplicate samples. (**e**) Degranulation of CD56^+^ NK cells was determined by FCM as the percentage of CD107a^+^ cells after overnight stimulation with different targets. Data are depicted as mean ± SD of triplicate samples. (**f**) IFNγ production was determined by ELISA and depicted as mean ± SD of triplicate measurements. (**g**) Specific cytotoxicity of a third CD56^+^ NK cell product (97% purity) against primary AML cells from 5 different patients. Specific lysis was determined after 24, 48 and 72 hours of co-culture in a FCM-based cytotoxicity assay at an E∶T ratio of 3∶1. Data are displayed as mean ± SD of triplicate samples.

To determine the cytotoxic potential of the *ex vivo*-generated NK cell products, we performed flow cytometry-based cytotoxicity assays using various myeloid leukemia cell lines (K562, Lama, Kasumi and KG1a), primary AML cells and two melanoma cell lines (BLM and FM3). *Ex vivo*-generated NK cells in GBGM® mediated efficient lysis of K562 cells (∼40% and ∼90% after 4 and 24 hrs, respectively) at a very low E∶T ratio of 2∶1 (Figure 6a). Furthermore, profound lysis could be observed against MHC class I-expressing KG1a cells (∼20% and ∼40% after 4 and 24 hrs, respectively). Interestingly, NK cells cultured in GBGM® showed higher cytotoxic and degranulating activity as compared to NK cells cultured in H3000 medium from the same UCB donor (Figure S5a–c). Furthermore, we found that GBGM-derived NK cells showed higher expression of the activating receptors NKG2D, NKp30, NKp44 and NKp46 (Figure S5d).

In addition to effective lysis of K562, also the leukemia cell lines Lama and Kasumi as well as the BLM and FM3 melanoma cells were efficiently lysed by the NK cells (Figure 6d). Interestingly, a monolayer of FM3 melanoma cells was completely destroyed within one hour of co-culture with NK cells (Movie S1). High NK cell-mediated cytolytic activity against leukemia and melanoma cell lines was associated with a high percentage of CD107a^+^ NK cells (Figure 6b and 6e) and significant production of IFNγ (Figure 6c and 6f).

Finally, we investigated whether primary leukemia cells are susceptible to killing by the *ex vivo*-generated NK cells. Therefore, we performed cytotoxicity assays with AML cells from five different patients. Primary AML cells from three patients (Pt 2, 4 and 5) were substantially killed within 3 days of co-culture at a low E∶T ratio of 3∶1 albeit less potent than K562 cells (Figure 6g). These results indicate that UCB-derived NK cells generated by our efficient culture method have the ability to kill myeloid leukemia and solid tumor cells.

### *Ex vivo* Generation of NK Cell Products from Frozen CD34^+^ UCB Cells

To investigate whether our NK cell expansion protocol could be adapted to a clinically applicable procedure, we have performed experiments using GBGM® medium in which we left out LIF and MIP-1α in the low-dose cytokine mixture because these cytokines are not available clinical grade. Furthermore, we performed these experiments using CD34^+^ cells selected from thawed UCB according to Method III depicted in Figure 1. Thawing and CD34^+^ cell selection resulted in obtaining 1.30±0.61×10^6^ (range 0.84–2.50×10^6^) CD34^+^ UCB cells from six donors (Table 2). *Ex vivo* generation using Method III resulted in a calculated NK cell yield of 4.6±2.4×10^9^ (range 1.9–7.8×10^9^) NK cells with a purity of >95% after 6 weeks of culture (Table 2). The expansion rate ranged between 1,500 and 6,500 fold starting with CD34^+^ cells from cryopreserved UCB. These data demonstrate that the transfer of our described procedure to clinical applicable conditions is feasible, and generates pure NK cell products with a more than 3-log expansion potential.

**Table 2:** Overview of CD34+ cell selections and NK cell culturing procedures.

			Method I - H3000		Method I - Stemline I		Method I - Stemline II	
	CD34 (%)	CD34 cells (×10^6^)	CD56 (%)	CD56 cells (×10^9^)	CD56 (%)	CD56 cells (×10^9^)	CD56 (%)	CD56 cells (×10^9^)
UCB1	78	1.10	50	0.57	74	1.20	87	2.20
UCB2	55	0.32	88	0.68	65	0.15	82	0.15
UCB3	93	0.52	94	3.10	43	0.55	71	0.50
UCB4	69	0.45	n.d.	n.d.	99	3.50	57	0.12
UCB5	87	0.20	n.d.	n.d.	75	0.40	48	0.02
UCB6	83	0.94	n.d.	n.d.	97	10.0	49	0.12
mean	78	0.59	77	1.50	75	2.70	66	0.51
SD	14	0.36	24	0.14	21	4.00	17	0.82
range	55–93	0.20–1.10	50–94	0.57–3.10	43–99	0.15–10.0	48–87	0.02–2.20
median	81	0.49	88	0.68	75	0.90	64	0.14

			Method II - H3000		Method II - Stemline I		Method II–GBGM	
	CD34 (%)	CD34 cells (×10^6^)	CD56 (%)	CD56 cells (×10^9^)	CD56 (%)	CD56 cells (×10^9^)	CD56 (%)	CD56 cells (×10^9^)
UCB7	68	0.50	91	11.0	40	0.69	98	7.7
UCB8	79	0.20	89	6.1	60	0.66	99	8.5
UCB9	96	0.24	85	3.4	88	3.20	100	18.0
mean	81	0.31	88	6.9	63	1.53	99	11.0
SD	14	0.16	3	4.0	24	1.50	1	5.5
range	68–96	0.20–0.50	85–91	3.4–11.0	40–88	0.69–3.20	98–100	7.7–18.0
median	79	0.24	89	6.1	60	0.69	99	8.5

			Method III - GBGM					
	CD34 (%)	CD34 cells (×10^6^)	CD56 (%)	CD56 cells (×10^9^)				
UCB10	98	0.84	99	5.5				
UCB11	96	1.20	96	1.9				
UCB12	95	1.00	97	2.0				
UCB13	88	0.92	95	4.0				
UCB14	98	1.30	97	6.6				
UCB15	98	2.50	97	7.8				
mean	96	1.30	97	4.6				
SD	4	0.61	1	2.4				
range	88–98	0.84–2.50	95–99	1.9–7.8				
median	97	1.10	97	4.7				

Table 2 shows a summary of all different experiments and the corresponding UCB donors using the different culture methods I–III and media (see Figure 1). The purity and the total number of CD34^+^ cells after immunomagnetic selection determined by FCM are depicted for each UCB donor. The CD56 content after 5 weeks (Method I and II) or 6 weeks (Method III) of culture was analyzed by FCM as described in Materials and Methods. Numbers of NK cells represent a theoretical calculation based on the actual expansion rates of CD56^+^ NK cells and were calculated using the initial numbers of CD34^+^ cells from each UCB multiplied by the NK cell expansion rate from each culture procedure. The mean ± standard deviation (SD), range and median are calculated for each method and medium separately.

## Discussion

Here, we report a novel and highly potent two-step culture method for the *ex vivo* generation of functional NK cells for clinical application in the treatment of patients with AML and other malignancies. We implemented a new clinical grade basal medium, designated GBGM®, which facilitates efficient *ex vivo* HPC and NK cell expansions. Our method enables the generation of functional human NK cells more than 4-logs from CD34^+^ cells enriched from freshly collected UCB units and more than 3-log from frozen UCB. To the best of our knowledge, this is the first demonstration that human CD56^+^ NK cells can be efficiently generated *ex vivo* at such high log-scale magnitude.

Similar studies have been reported previously using different combinations of cytokines with or without feeder cell lines and use of animal and human sera [15]–[17], [20]–[24]. For instance, a recent study by Kao et al. showed that serum-free expanded CD34^+^ cells could be differentiated into a NK cell product with an average purity of 40%–60% after 5–7 weeks of culture. They reached a calculated mean expansion rate of 300-fold, but used fetal bovine serum during the NK cell generation phase [16]. Compared to these recently published data, our novel culture system holds great promise, resulting in more than 3-log-scale *ex vivo* generation of NK cell products with nearly 100% purity using clinical grade medium, human serum, and cytokines. The only cytokines that are currently or in the near future not available as clinical grade reagents are LIF and MIP-1α, which are part of the low-dose supporting cytokine cocktail. However, experiments using CD34^+^ selected cells from cryopreserved UCB revealed that these two factors can be discarded without the loss of the NK cell differentiation potential of our refined protocol (Table 2). Taking the high expansion potential, our system allows the generation of an average number of 4.6×10^9^ highly pure NK cells from frozen CD34^+^ UCB cells (Table 2). Moreover, preliminary upscaling experiments in cell culture bags revealed that the established culture procedure generates up to 2×10^9^ NK cells with the same phenotype and function as shown for the NK cell populations generated in tissue culture plates (data not shown). These findings indicate that our refined *ex vivo* expansion protocol has the potential to produce more NK cells (i.e. >2×10^9^ with a purity between 95–99%) as compared to purification of mature NK cells from a 15 liter lymphapheresis procedure, which yields 0.25×10^9^±0.14×10^9^ (range 0.01–0.47×10^9^) NK cells according to the published data of McKenna et al. in 2007 [7]–[12].

The NK cell product generated by our method displayed reproducibly an activated phenotype with a high expression of CD56 and various activating receptors, such as NKG2D, NKp30, NKp44 and NKp46. In line with earlier observations, our *ex vivo*-generated NK cells cultured in the presence of IL-2 and IL-15 showed a CD56^bright^ phenotype of which only a subset expressed CD16 [20], [21], [25], [26]. The resulting NK cell products contained 40–60% CD56^+^NKG2A^+^ NK cells and up to 10% of the CD56^+^ population expressed various KIRs, though there was considerable variation in the frequency of KIR^+^ NK cell subsets between the different UCB donors. According to the NK cell differentiation model of Freud and Caligiuri, our *ex vivo*-generated NK cell products predominantly contain developmentally mature NK cells expressing high CD94/NKG2A, CD117 and/or KIR mediating potent cytolytic activity against K562 cells [27]. A minority of the CD56^+^ NK cells in our products are phenotypically immature lacking NKG2A and KIR. However, these immature NK cells may have the potential to maturate *in vivo* following adoptive transfer. Interestingly, Cooley et al. have shown that CD56^+^NKG2A^−^KIR^−^ NK cells are able to mature *in vitro* upon culture with IL-15 and a stromal feeder cell line [28]. Phenotypic analysis have revealed that UCB-derived NK cells generated with our protocol display expression of cytokine receptors including IL-2/IL-15Rβ (CD122), which may promote *in vivo* survival, expansion and maturation upon transfer following immunosuppressive therapy.

The strong cytotoxic activity of our UCB-derived NK cells against various tumor cell lines as well as the cytolysis of primary AML cells were displayed by specific lysis, CD107a-mediated degranulation and the production of IFNγ. Most experiments were done with AML cell lines and primary AML cells, which has been shown to be an attractive target for NK cell-mediated immunotherapy [5], [6]. Interestingly, we also observed efficient lysis of melanoma cell lines, which strengthens the therapeutic potential of our NK cells product also towards non-hematological cancers. Most recently, several studies demonstrated, that NK cell-mediated cytotoxicity and the use of NK cell-based immunotherapy could be an efficient approach for the treatment of melanoma, which was also demonstrated in a phase I trial using the NK-92 cell line [29]–[31].

In conclusion, the method presented here provides an important advance for generating clinically relevant NK cell products from hematopoietic stem and progenitor cells with high cell numbers, high purity and functionality for use in NK cell-based immunotherapy. These *ex vivo*-generated NK cell products can be exploited for adoptive immunotherapy either following haploidentical SCT for boosting NK cell-mediated graft-versus-leukemia reactivity or in the non-transplant setting following lymphodepleting immunosuppressive regimens. Our current NK cell generation protocol has been optimized for the use of CD34^+^ cells from UCB, which can be readily obtained from cord blood banks. Furthermore, the described modifications of our protocol and use of the GBGM® medium have also enabled the generation of NK cells from bone marrow or mobilized peripheral blood CD34^+^ cells (preliminary data not shown). Currently, our first aim is to explore the feasibility of adoptive transfer of *ex vivo*-generated NK cell products from KIR-ligand mismatched UCB donors in elderly patients with AML following an intensive immunosuppressive regimen. Important aspects to study in this trial are whether *ex vivo*-generated NK cell products are able to survive, migrate and expand *in vivo* following infusion into preconditioned patients.

## Materials and Methods

### Cell Lines

Cell lines (K562, KG1a, Lama, Kasumi, BLM and FM3) were cultured in Iscove's modified Dulbecco's medium (IMDM; Invitrogen, Carlsbad CA, USA) containing 50 U/ml penicillin, 50 µg/ml streptomycin and 10–20% fetal calf serum (FCS; Integro, Zaandam, The Netherlands) Characteristics of the cell lines used in functional assays are shown in Table 1. Clonal K562 cell lines expressing HLA-Cw*0301 and/or HLA-Cw*0403 were used as target cells to analyze specificity of the UCB-derived NK cells. Briefly, K562 cells were transfected with full length HLA-Cw0301 cDNA inserted into the EcoRI and HindIII sites of expression vector pcDNA3.1(−) (Invitrogen, Paisley, UK) and HLA-Cw*0403 cDNA ligated into the pEF6/V5-His expression vector using the TOPO TA Expression kit (Invitrogen). The cell lines K562-Cw*0301, K562-Cw*0403 and K562-Cw*0301/0403 showed stable and strong HLA class I expression (>95%) analyzed by flow cytometry (FCM) using the HLA class I antibody W6/32 (Sigma, Steinheim, Germany). The (co-)expression of HLA-Cw*0301 and HLA-Cw*0403 was confirmed by sequencing of HLA-Cw cDNA.

### Selection of CD34-Positive Stem and Progenitor Cells

UCB units have been obtained at birth after normal full-term delivery with written informed consent with regard of scientific use from the cord blood bank of the Radboud University Nijmegen Medical Center (RUNMC). UCB samples were stored at room temperature and processed within 24 h after collection. Mononuclear cells (MNC) were isolated by Ficoll-Hypaque (1.077 g/ml; GE Healthcare, Uppsala, Sweden) density gradient centrifugation. Alternatively, UCB samples were thawed at 37°C and resuspended in CliniMACS buffer (Miltenyi Biotech, Bergisch Gladbach, Germany) containing 5% HSA, 3.5 mM MgCl_2_ and 100 U/ml Pulmozyme (Genentech). After 30 min of incubation, thawed UCB cells were washed and used for CD34^+^ cell selection. CD34^+^ cells were selected from UCB cells using anti-CD34 immunomagnetic bead separation (Miltenyi Biotech, Bergisch Gladbach, Germany) according to manufacturer's instructions. The cell number and purity of the enriched CD34^+^ fraction was analyzed by FCM. Purity of the obtained cell populations was 85±13%.

### *Ex vivo* Expansion of CD34-Positive Progenitor Cells

CD34^+^ UCB cells (between 1×10^4^ and 3×10^5^ per ml) were plated into 24-well tissue culture plates (Corning Incorporated, Corning, NY) using Methods I, II or III (Figure 1) in the following basal media: StemSpan® H3000 (Stemcell Technologies, Grenoble, France), Stemline I™ and Stemline II™ Hematopoietic Stem Cell Expansion Medium (Sigma-Aldrich, Zwijndrecht, The Netherlands) and Glycostem Basal Growth Medium (GBGM®) (Clear Cell Technologies, Beernem, Belgium). H3000 and Stemline media were supplemented with 20 mg/ml ascorbic acid, 50 µmol/l ethanolamine, 50 µmol/l sodium selenite, 25 µmol β-mercaptoethanol (all Sigma Aldrich), 100 U/ml penicillin, 100 µg/ml streptomycin and 2 mmol/L L-glutamine (all Invitrogen). All media used in Method I and II were supplemented with 10% human serum (HS; Sanquin Bloodbank, Nijmegen, The Netherlands) and a low-dose cytokine cocktail consisting of 10 pg/ml GM-CSF, 250 pg/ml G-CSF, 50 pg/ml LIF, 200 pg/ml MIP-1α (all Stemcell Technologies) and 50 pg/ml IL-6 (CellGenix, Freiburg, Germany), which was based on studies using the fetal liver-derived stromal cell line AFT024 [32]. In Method III, LIF and MIP-1α were left out of the low-dose cytokine mixture. In addition, a high-dose cytokine cocktail was added consisting of 27 ng/ml SCF (CellGenix), 25 ng/ml Flt3L (CellGenix), 25 ng/ml TPO (Stemcell Technologies) and 25 ng/ml IL-7 (Stemcell Technologies). During the first 14 days of culture, low molecular weight heparin (LMWH) (Clivarin®; Abbott, Wiesbaden, Germany) was added to the expansion medium in a final concentration of 25 µg/ml. In Method II and III, TPO was replaced by 20 ng/ml IL-15 at day 9–14. Cell cultures were refreshed with new medium every 2–3 days. Cultures were maintained in a 37°C, 95% humidity, 5% CO_2_ incubator.

### Differentiation of *ex vivo* Expanded CD34-Positive Cells into NK Cells

Expanded CD34^+^ UCB cells were differentiated and further expanded using NK cell differentiation medium. This medium consisted of the same basal medium as used for the CD34 expansion step supplemented with 10% HS, the low-dose cytokine cocktail (as previously mentioned) and a new high-dose cytokine cocktail consisting of 20 ng/ml IL-7, 22 ng/ml SCF, 1000 U/ml IL-2 (Proleukin®; Chiron, München, Germany) and 20 ng/ml IL-15 (CellGenix). Flt3L (20 ng/ml) was only added to the differentiation medium in Method I (Figure 1). Medium was refreshed twice a week from day 14 onwards. Total and CD56^+^ cell expansion at each week of culture was calculated by the number of cultured cells divided by the number of seeded cells. The number of seeded cells was reduced by dilution with fresh differentiation medium. The theoretical total cell numbers were calculated by multiplying the expansion rate per week with the number cultured cells.

### Flow Cytometry

Cell numbers and expression of cell-surface markers were determined by FCM. Briefly, cells were incubated with the appropriate concentration antibodies for 30 min at 4°C. After washing, cells were resuspended in Coulter® Isoton® II Diluent (Beckman Coulter) and analyzed using the Coulter FC500 flow cytometer (Beckman Coulter). Cell numbers and NK cell purity have been determined by gating on CD45^+^ cells in combination with forward scatter (FS) and side scatter (SS). For phenotypic analysis of the NK cell products, living cells were gated only on FS/SS and further analyzed with the specific antigen of interest. The following conjugated antibodies were used: anti-CD16-FITC (NKP15), anti-CD94-FITC (HP-3D9), anti-NKG2D-PE (1D11) (BD Biosciences Pharmingen, Breda, The Netherlands), anti-CD161-PE (191B8), anti-NKG2A-PE (Z199), anti-CD122-PE (CF1) (Immunotech, Marseille, France), anti-NKG2C-PE (134591), anti-NKp80-PE (239127) (R&D System, Abingdon, UK), anti-CD45-ECD (J33), anti-CD117-PE-Cy5 (104D2D1), anti-CD34-PE-Cy7 (581), anti-CD56-PE-Cy7 (N901), anti-NKp30-PE (Z25), anti-NKp44-PE (Z231), anti-NKp46-PE (BAB281), anti-CD158a,h-PE (EB6.B), anti-CD158b1/b2-PE (GL183), anti-CD158e1/e2-PE (Z27), anti-CD158i-PE (FES172), anti-CD3-ECD (UCHT1), anti-CD244-PE (C1.7), anti-CD25-PE-Cy5 (B1.49.9), anti-CD7-PECy5 (8H8.1), anti-CD2-FITC (39.C1.5) (all Beckman Coulter, Woerden, The Netherlands) and anti-HLA-ABC-PE (W6/32) (Dako, Enschede, The Netherlands).

### Flow Cytometry-Based Cytotoxicity and Degranulation Studies

FCM-based cytotoxicity assays were performed as described previously [33] with minor adaptations. Target cells were labeled with 0.5 µM carboxyfluorescein diacetate succimidyl ester (CFSE; Molecular Probes Europe, Leiden, The Netherlands) in a concentration of 1×10^7^ cells per ml for 10 min at 37°C. The reaction was terminated by adding an equal volume of FCS, followed by incubation at room temperature for 2 min and stained cells were washed twice with 5 ml IMDM/10% FCS. After washing, cells were resuspended in IMDM/10% FCS to a final concentration of 2×10^5^/ml. CD56^+^ NK cells were washed with PBS and resuspended in IMDM/10% FCS to a final concentration of 2×10^5^/ml. Target cells (2×10^4^) were co-cultured with effector cells at different E∶T ratio's in a total volume of 200 µl IMDM/10% FCS in 96-wells flat-bottom plates. NK cells and target cells alone were plated out in triplicate as controls. NK cell co-cultures with primary AML cells were supplemented with IL-3 (50 ng/ml), SCF (25 ng/ml), Flt3L (20 ng/ml), GM-CSF (100 ng/ml) and G-CSF (100 ng/ml). To measure degranulation by NK cells, anti-CD107a (H4A3; BD Biosciences) was added in a 1∶200 dilution to the co-cultures. After incubation for 4 or 24 h at 37°C, 50 µl supernatant was collected and stored at −20°C for later use to measure cytokine production. Cells in the remaining volume were harvested and the number of viable target cells was quantified by FCM. Target cell survival was calculated as follows: % survival  =  {[absolute no. viable CFSE+ target cells co-cultured with NK cells]/[absolute no. viable CFSE+ target cells cultured in medium]}*100%. The percentage specific lysis was calculated as follows: % lysis  =  {100−[% survival]}. Degranulation of NK cells during co-culture was measured by cell surface expression of CD107a [34]. After 2, 4 or 24 hrs of incubation at 37°C, the percentage of CD107a+ cells was determined by FCM.

### IFNγ Production Assay

Production of IFNγ by target cell-stimulated NK cells was measured in the supernatant of the co-cultures by ELISA (Pierce Endogen, Rockford, IL, USA). Absorbance was measured at 450 nm with a Multiscan MCC/340 ELISA reader (Titertek, Huntsville, Alabama, USA).

### Life Imaging NK Cell-Mediated Killing

NK cell-mediated killing of solid tumor cell lines was visualized by life imaging on a Zeiss Axiovert 35 M inverted contrast microscope (Zeiss, Sliedrecht, The Netherlands) which was placed in a 37°C incubator. Images were digitized in 768×512 pixels with a camera (VarioCam, PCO computer Optics GmbH, Kelheim, Germany), coupled with the Pixel Pipeline in a Macintosh-G4. Video recordings of microscopic fields were made continuously (1 image/min) using an in house developed software program version of the IPlab 3.5.5. image program (Scananlytics Inc. USA All). Images were further processed using the NIH Image 1.61 program, resulting in a Quick-Time movie.

### Statistics

Results from different experiments are described as mean ± standard deviation of the mean (SD). Statistical analysis was performed using ANOVA and Duncan Post-hoc comparison. A p-value of <0.05 was considered statistically significant.

## Supporting Information

Figure S1 Expansion of CD34-enriched UCB cells using a cytokine-based culture method. CD34^+^ UCB cells were selected by immunomagnetic beads and cultured for 2 weeks in three different basal media supplemented with 10% HS, a low-dose cell supporting cytokine cocktail, a high-dose cell expansion cocktail and clinical grade low molecular weight heparin (see details in Materials and Methods and Figure 1). Absolute CD34^+^ cell numbers (a), fold expansion of total cells (b) and CD34 content (c) were determined by FCM after one and two weeks of culture using Method I. CD34^+^ cell numbers and fold expansion of total cells using Method II (d+e). Data are depicted as mean ± SD for the different media, which have been tested in parallel experiments with CD34^+^ cells from 3–6 UCB donors.
(6.73 MB TIF)

Figure S2 Phenotypical profile of *ex vivo*-generated NK cells using Method II with GBGM. (a) Flow cytometric analysis of a second NK cell product generated from CD34^+^ UCB progenitor cells. Cells at 5 weeks of culture were analyzed for expression of CD56, CD3, CD94 and CD16. (b) Expression of a repertoire of receptors important for regulating NK cell activity, including C-type lectin receptors, natural cytotoxicity receptors and cytokine receptors. Histograms show expression of the antigen of interest (black histogram) compared to the specific isotype control (grey histogram). (c) Acquisition of KIR^+^ NK cell subsets during *ex vivo* NK cell generation from expanded CD34^+^ UCB cells. KIR expression was determined at week 4 and 5 during the differentiation step by FCM.
(7.08 MB TIF)

Figure S3 Responsiveness of *ex vivo*-generated KIR^+^ NK cells generated using Method II with GBGM to MHC class I-deficient target cells. *Ex vivo*-generated NK cells were incubated alone, or 18 hours with MHC class I-negative K562 or MHC class I-expressing KG1a cells at an E∶T ratio of 1∶1. Cells were then stained for CD56, CD3, KIR, and the degranulation antigen CD107a. Shown are the degranulation of total CD56^+^CD3^−^ NK cells and KIR2DL2/DL3^+^ NK cell subset expanded for 5 weeks from CD34^+^ UCB cells. Density plots are gated on CD56^+^CD3^−^ NK cells and the histogram plots show the CD107a degranulation of the KIR2DL2/DL3^+^ NK cells.
(5.06 MB TIF)

Figure S4 Responsiveness of *ex vivo*-generated KIR^+^ NK cells generated by Method II to target cells expressing different KIR ligands. Activity of UCB-derived NK cells derived from two different donors were tested in a 2 hour CD107a degranulation assay against K562 cells transfected with empty vector (EV), HLA-Cw3 cDNA, HLA-Cw4 cDNA or both.
(7.22 MB TIF)

Figure S5 Functional activity of *ex vivo*-generated CD56^+^ NK cells using Method II. (a) Specific cytotoxicity of two CD56^+^ NK cell products from the same UCB donor but cultured either in H3000 or GBGM against the myeloid leukemia cell lines K562 and KG1a. Specific lysis was determined after 24 hours of co-culture in a FCM-based cytotoxicity assay at an E∶T ratio of 2∶1. Data are displayed as mean ± SD of triplicate wells. (b) Degranulation of CD56^+^ NK cells was determined by FCM as the percentage of CD107a^+^ cells. Results are depicted as mean ± SD of triplicate wells. (c) IFNγ production was determined by ELISA and depicted as mean ± SD of triplicate measurements. (d) Expression of activating receptors important for NK cell activity. Histograms show antigen expression of GBGM-derived NK cells (black histogram) compared to H3000-derived NK cells (grey histogram) and the specific isotype control (white histogram).
(8.74 MB TIF)

Movie S1 Movie showing the strong killing potential of *ex vivo*-generated NK cells (bright spots) towards the adherent growing melanoma cell line FM3 within one hour of co-culture. NK cells were added to the melanoma tumor cell layer at an E∶T ratio of 1∶1.
(1.43 MB AVI)
