# Supplementary figures and images for: High Log-Scale Expansion of Functional Human Natural Killer Cells from Umbilical Cord Blood CD34-Positive Cells for Adoptive Cancer Immunotherapy
Source: PLoS One. 2010 Feb 15;5(2):e9221. doi: 10.1371/journal.pone.0009221 (PMC2821405; doi:10.1371/journal.pone.0009221)

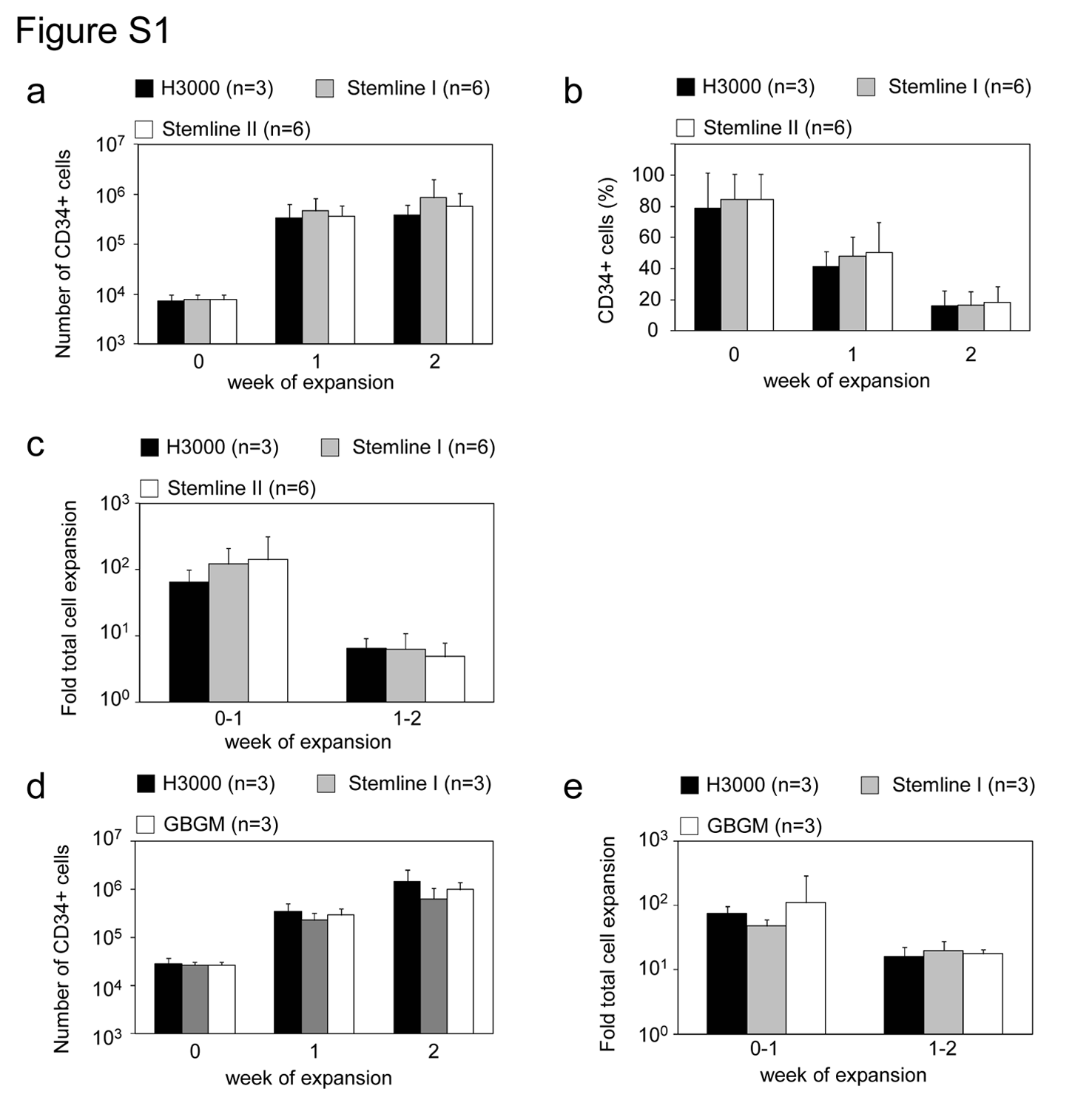

Supplement: Figure S1 — Expansion of CD34-enriched UCB cells using a cytokine-based culture method. CD34+ UCB cells were selected by immunomagnetic beads and cultured for 2 weeks in three different basal media supplemented with 10% HS, a low-dose cell supporting cytokine cocktail, a high-dose cell expansion cocktail and clinical grade low molecular weight heparin (see details in Materials and Methods and Figure 1). Absolute CD34+ cell numbers (a), fold expansion of total cells (b) and CD34 content (c) were determined by FCM after one and two weeks of culture using Method I. CD34+ cell numbers and fold expansion of total cells using Method II (d+e). Data are depicted as mean ± SD for the different media, which have been tested in parallel experiments with CD34+ cells from 3–6 UCB donors. (6.73 MB TIF) [file pone.0009221.s001.tif]

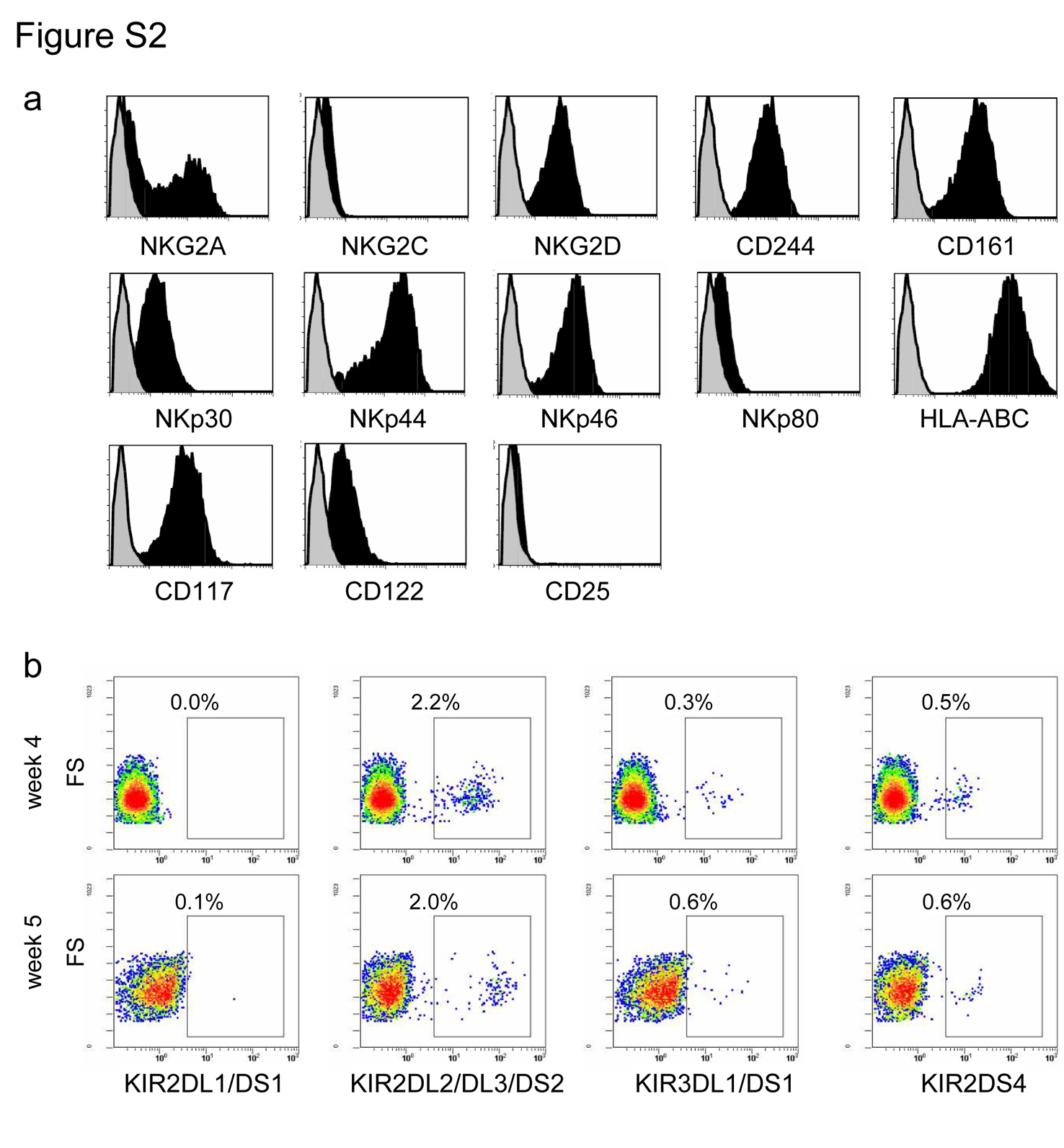

Supplement: Figure S2 — Phenotypical profile of ex vivo-generated NK cells using Method II with GBGM. (a) Flow cytometric analysis of a second NK cell product generated from CD34+ UCB progenitor cells. Cells at 5 weeks of culture were analyzed for expression of CD56, CD3, CD94 and CD16. (b) Expression of a repertoire of receptors important for regulating NK cell activity, including C-type lectin receptors, natural cytotoxicity receptors and cytokine receptors. Histograms show expression of the antigen of interest (black histogram) compared to the specific isotype control (grey histogram). (c) Acquisition of KIR+ NK cell subsets during ex vivo NK cell generation from expanded CD34+ UCB cells. KIR expression was determined at week 4 and 5 during the differentiation step by FCM. (7.08 MB TIF) [file pone.0009221.s002.tif]

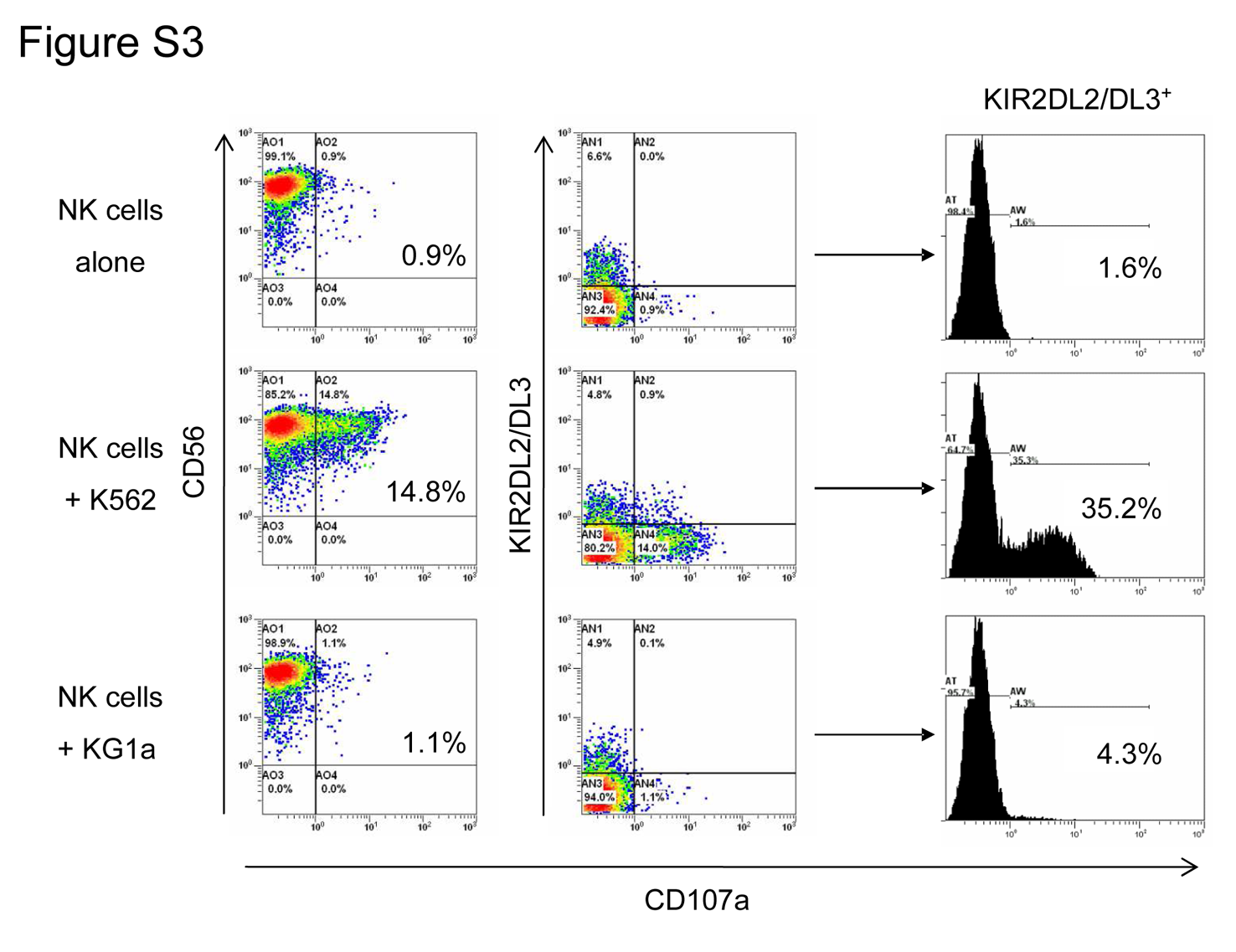

Supplement: Figure S3 — Responsiveness of ex vivo-generated KIR+ NK cells generated using Method II with GBGM to MHC class I-deficient target cells. Ex vivo-generated NK cells were incubated alone, or 18 hours with MHC class I-negative K562 or MHC class I-expressing KG1a cells at an E∶T ratio of 1∶1. Cells were then stained for CD56, CD3, KIR, and the degranulation antigen CD107a. Shown are the degranulation of total CD56+CD3− NK cells and KIR2DL2/DL3+ NK cell subset expanded for 5 weeks from CD34+ UCB cells. Density plots are gated on CD56+CD3− NK cells and the histogram plots show the CD107a degranulation of the KIR2DL2/DL3+ NK cells. (5.06 MB TIF) [file pone.0009221.s003.tif]

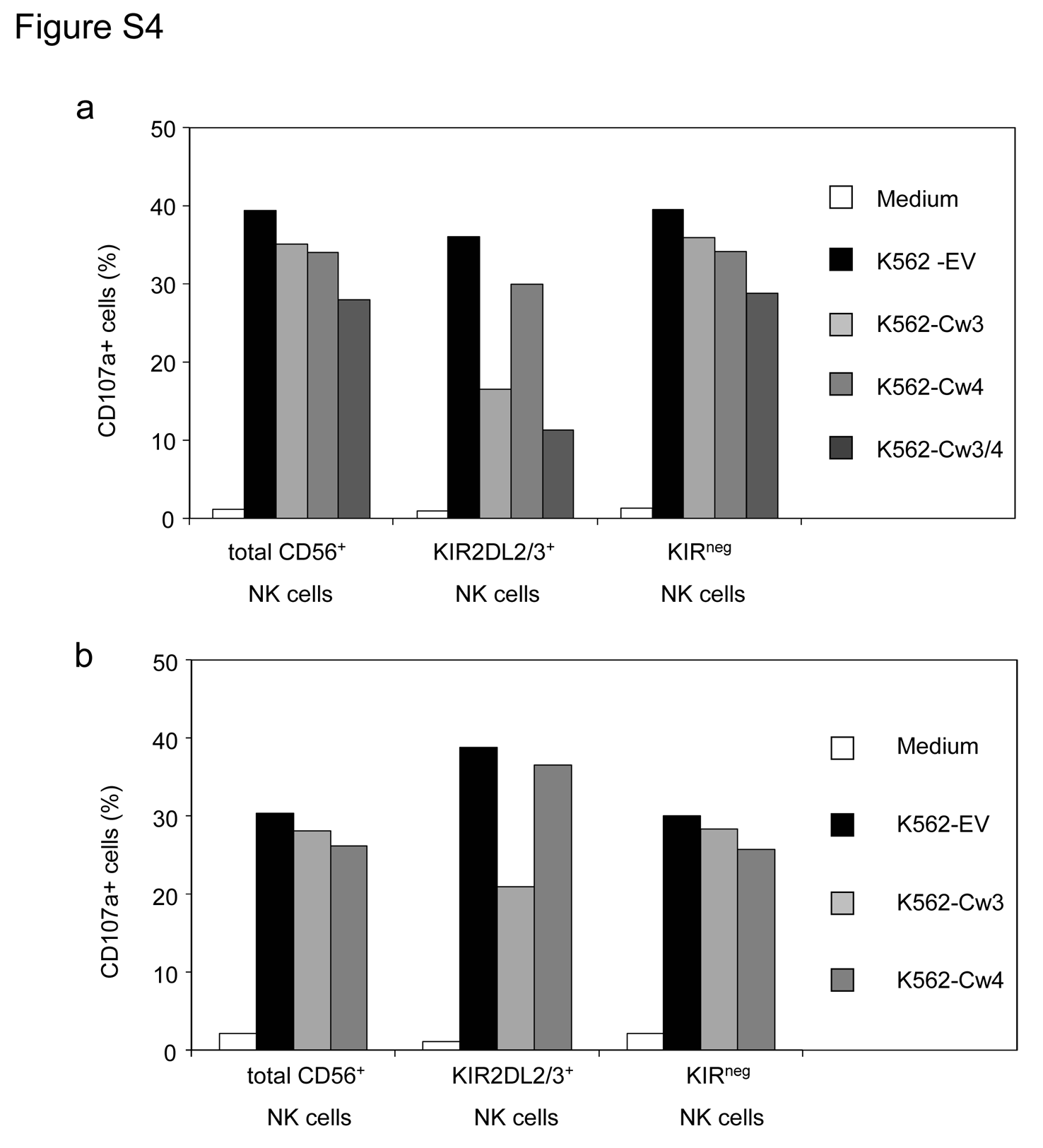

Supplement: Figure S4 — Responsiveness of ex vivo-generated KIR+ NK cells generated by Method II to target cells expressing different KIR ligands. Activity of UCB-derived NK cells derived from two different donors were tested in a 2 hour CD107a degranulation assay against K562 cells transfected with empty vector (EV), HLA-Cw3 cDNA, HLA-Cw4 cDNA or both. (7.22 MB TIF) [file pone.0009221.s004.tif]

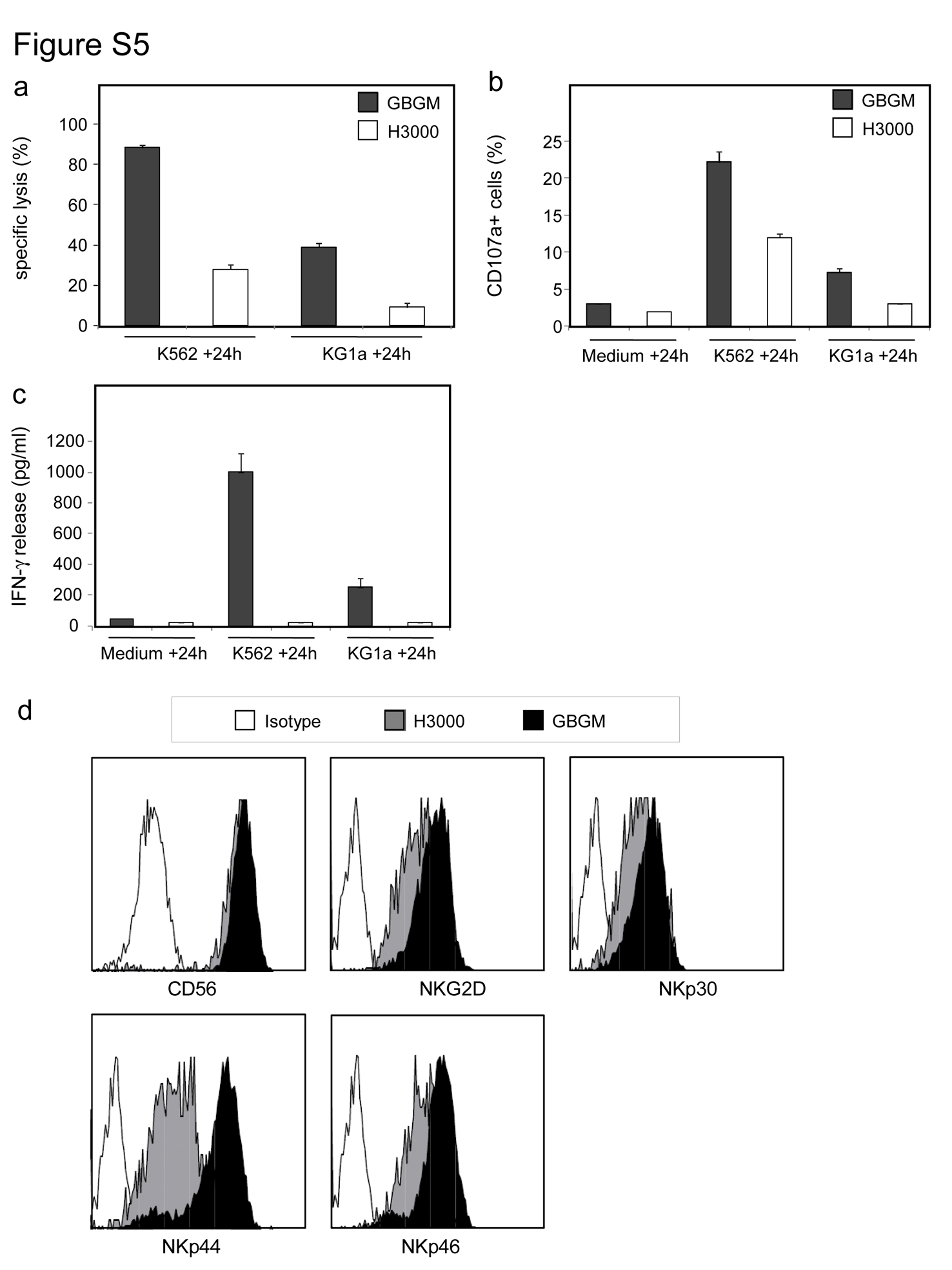

Supplement: Figure S5 — Functional activity of ex vivo-generated CD56+ NK cells using Method II. (a) Specific cytotoxicity of two CD56+ NK cell products from the same UCB donor but cultured either in H3000 or GBGM against the myeloid leukemia cell lines K562 and KG1a. Specific lysis was determined after 24 hours of co-culture in a FCM-based cytotoxicity assay at an E∶T ratio of 2∶1. Data are displayed as mean ± SD of triplicate wells. (b) Degranulation of CD56+ NK cells was determined by FCM as the percentage of CD107a+ cells. Results are depicted as mean ± SD of triplicate wells. (c) IFNγ production was determined by ELISA and depicted as mean ± SD of triplicate measurements. (d) Expression of activating receptors important for NK cell activity. Histograms show antigen expression of GBGM-derived NK cells (black histogram) compared to H3000-derived NK cells (grey histogram) and the specific isotype control (white histogram). (8.74 MB TIF) [file pone.0009221.s005.tif]
